# Histomorphometric analysis of the human internal thoracic artery and relationship with cardiovascular risk factors

**DOI:** 10.1371/journal.pone.0211421

**Published:** 2019-01-25

**Authors:** Diogo A. Fonseca, Pedro E. Antunes, Manuel J. Antunes, Maria Dulce Cotrim

**Affiliations:** 1 Laboratory of Pharmacology and Pharmaceutical Care, Faculty of Pharmacy, University of Coimbra, Coimbra, Portugal; 2 Coimbra Institute for Clinical and Biomedical Research (iCBR), Faculty of Medicine, University of Coimbra, Coimbra, Portugal; 3 Centre of Cardiothoracic Surgery, University Hospital and Faculty of Medicine of Coimbra, Coimbra, Portugal; University of Bologna, ITALY

## Abstract

In this study, we aimed at performing a histomorphometric analysis of human left internal thoracic artery (ITA) samples as well as at correlating the histomorphometric findings with the clinical profile, including risk factors and medication. Distal segments of ITA were obtained from 54 patients undergoing coronary artery bypass grafting. Histological observation was performed in paraffin-embedded transverse sections of ITA through four staining protocols: hematoxylin-eosin, van Gieson, Masson’s trichrome and von Kossa. Morphometric analysis included the intimal width (IW), medial width (MW) and intima/media ratio (IMR). No overt atherosclerotic lesions were observed. Mild calcifications were observed across the vascular wall layers in almost all samples. Multivariable linear regression analysis showed associations between IW and IMR and the following clinical variables: age, gender, kidney function expressed as eGFR and myocardial infarction history. Age (odds ratio = 1.16, *P* = 0.004), female gender (odds ratio = 11.34, *P* = 0.011), eGFR (odds ratio = 1.03, *P* = 0.059) and myocardial infarction history (odds ratio = 4.81, *P* = 0.040) were identified as the main clinical predictors for intimal hyperplasia. Preatherosclerotic lesions in ITA samples from patients undergoing coronary revascularization were associated not only with classical cardiovascular risk factors such as age and gender, but also with other clinical variables, namely kidney function and myocardial infarction history.

## Introduction

Vascular structural changes have been associated with cardiovascular diseases [1]. In particular, carotid intima-media thickness (IMT) is associated with risk factors [2] and is an independent predictor for cardiovascular outcomes [1, 3–5]. Furthermore, Iwamoto et al. [1] suggested the use of the brachial IMT as a marker for the atherosclerosis grade. In this study, the authors also showed that increased brachial IMT was associated with decreased flow-mediated dilation, thus suggesting a strong connection between the structural and functional properties of the vascular system.

A wide array of techniques is available to study the vascular histomorphology. Despite the growing use of non-invasive methods, the histomorphologic study of isolated vessels remains as a useful tool to understand the structural properties of vessels. Moreover, the quantitative assessment of the vascular structure provides valuable information that complements the qualitative microscopical observation [6]. Several histomorphometric parameters have been reported in the literature, namely: maximal intimal width (IW), medial width at maximal intimal width (MW), intimal and medial areas and others [6]. Additional parameters may be calculated from these primary parameters, particularly intima/media ratio (IMR), which has been suggested as the most sensitive method for grading atherosclerosis and intimal hyperplasia [7, 8].

The human internal thoracic artery or ITA (also known as internal mammary artery) has long been recognized as atherosclerosis-resistant vessel [9–11]. Several reports have provided distinct evidence in regard to the relationship between the structural properties of the ITA and cardiovascular risk factors, as previously reviewed by us [6].

In this context, we aimed at performing a histomorphometric analysis of ITA samples and at correlating the histomorphometric findings with the clinical profile, including risk factors and medication.

## Materials and methods

### Ethical approval

Experiments were performed on left ITA distal samples harvested from patients undergoing coronary revascularization. Written informed consent was obtained from each patient and the experiments were performed with the approval from the research ethics committees of the Faculty of Medicine of University of Coimbra and the University Hospital of Coimbra (Coimbra, Portugal), with the following references CE-107/2014 and PC-388/08, respectively. The study was conducted in accordance with the Declaration of Helsinki and was not registered in any research database.

### Clinical variable definition

Smoking history was defined as history of consumption of any form of tobacco (cigarettes, cigars, tobacco chew, smoking pipe or others). Arterial hypertension was defined as systolic and diastolic blood pressure exceeding 140 mmHg and 90 mmHg, respectively, or history of high blood pressure or need of antihypertensive drugs. Diabetes mellitus was defined as history of diabetes and current treatment with either insulin or oral drugs. Dyslipidemia was defined as the presence or absence of history of dyslipidemia diagnosed and/or treated by a physician. Peripheral vascular disease (PVD) was defined as claudication either with exertion or at rest; amputation for arterial insufficiency; aorto-iliac occlusive disease reconstruction; peripheral vascular bypass surgery, angioplasty or stent; documented abdominal aorta aneurysm, repair or stent; or non-invasive carotid test with > 75% occlusion. Cerebrovascular disease was defined as unresponsive coma for longer than 24h, cerebrovascular accident or transient ischemic attack. Kidney function was evaluated by the value of estimated glomerular filtration rate (eGFR), from Modification of Diet in Renal Disease formula [12]. The highest serum level of creatinine within 2 days preceding the surgery was taken as the preoperative creatinine level. In terms of preoperative medication, we included the following drug classes: angiotensin converting enzyme inhibitors (ACEI), angiotensin II receptor blockers (ARB), β-blockers, calcium channel blockers (CCB), insulin, oral hypoglycemic agents (OHA) and nitrates.

### Vessel harvesting and preparation

Vessel samples from 54 patients were harvested and prepared as described previously [13]. Briefly, ITAs were harvested as a pedicle after sternal incision and were externally irrigated with papaverine to prevent vasospasm. Distal portions of ITA discarded from surgery were placed in cold (4°C) Krebs-Henseleit bicarbonate buffer (in mM: 118.7 NaCl, 5.4 KCl, 1.9 CaCl_2_.2H_2_O, 0.9 KH_2_PO_4_, 0.6 MgSO_4_.7H2O, 25 NaHCO_3_ and 11.1 Glucose), previously aerated with 95% O_2_ / 5% CO_2_ and adjusted to pH 7.4, and then isolated to remove most of the perivascular tissue.

### Histomorphologic evaluation

Left ITA samples were fixated in 10% buffered formaldehyde. Histomorphology was observed with a light microscope (Leica DM1000 LED). Samples were studied with the following stainings: (a) Hematoxylin-Eosin, (b) Verhoeff-Van Gieson (VVG, to study the elastic elements), (c) Masson’s Trichrome (to observe the collagen and muscle content of the vessel wall) and (d) von Kossa (to observe calcifications). Staining protocols from the manufacturer were followed and performed in the Laboratory of Experimental Pathology, Faculty of Medicine, University of Coimbra.

Histomorphologic analysis included both histopathologic and histomorphometric analysis. For the purposes of histomorphometric analysis, the Java-based image processing software ImageJ was used. Calibration of the scale in ImageJ was performed considering the distance per pixel indicated for each image by the Leica Application Suite V4.8.0 (Leica Microsystems). Maximal IW and MW were measured and mean values of 2 measurements per location were determined. Furthermore, IMR was calculated as the ratio between IW and MW. These results were then stratified according to the classification previously proposed by Kaufer et al. [14]: grade 0 (IMR ≤ 0.25), grade 1 (0.25 < IMR ≤ 0.5), grade 2 (0.5 < IMR ≤ 0.75) and grade 3 (IMR > 0.75).

### Analysis of results

Data is generally presented as mean ± standard error of mean (SEM) for continuous variables and as frequencies and percentages for categorical variables. N corresponds to the Number of patients.

Multivariable linear and logistic regression analysis were performed to identify clinical predictors for continuous and dichotomous dependent variables, respectively. Clinical variables with a significant individual association in analysis of variance (P < 0.200) were retained for regression analysis by the backward stepwise method.

For the linear regression models, the adjusted R^2^ is presented as a measure of percentage of explanation of the models (e.g. an adjusted R^2^ of 0.200 means that 20.0% of the variation may be explained by the variables included in the model). Constructed model presented higher predictive power than no model as assessed by analysis of variance with P < 0.050. Multicollinearity was assessed by the variance inflation factor (VIF).

For the logistic regression model, the model performance was evaluated considering two properties, (a) the calibration and (b) the discriminatory power. Calibration was assessed by the Hosmer-Lemeshow test which analyses the differences between the observed and the predicted results. The obtainment of a non-significant *p* value (P > 0.050) indicates a good calibration of the model [15].

The discriminatory power was evaluated considering the area under the receiver operating characteristic (ROC) curve or AUC [16], which was obtained by the nonparametric approach of Wilcoxon-Mann-Whitney suggested by Hanley and McNeil [17]. If the AUC is superior to 0.7, we may consider that the model presents a satisfactory discriminatory power [18]. The R^2^ is also presented as a measure of percentage of explanation of the model.

Statistical analysis was performed with IBM SPSS Statistics version 25.0.0 (IBM Corp., Armonk, NY, USA) and JMP Pro 13.1.0 (SAS Institute Inc., Cary, NC, USA) and graphs were prepared with GraphPad Prism 7 (GraphPad Software, Inc., La Jolla, CA, USA).

### Drugs used

Verhoeff’s Elastic Van Gieson Stain Kit (RRSK40), Masson Trichrome Stain Kit (RRSK20) and Von Kossa Stain Kit (RRSK39) were purchased from Atom Scientific. All other chemicals were purchased from Sigma-Aldrich (St. Louis, Missouri, USA) and correspond to the highest grade commercially available.

## Results

### Baseline characteristics of population

A total of 54 patients were included in this study (Table 1). The average age was 65.4 ± 1.4 years. The majority of the patients were male (81.5%) and arterial hypertension (87.0%) and dyslipidemia (87.0%) were the most prevalent comorbidities. Smoking history was only observed in male group (Table 1). Moreover, 24 patients (44.4%) had myocardial infarction history.

**Table 1:** Clinical characteristics of total population and according to gender. Continuous variables presented as mean ± SEM and categorical variables as counts (percentages).

Variable	Total (n = 54)	Females (n = 10)	Males (n = 44)	P
Age (years)	65.4 ± 1.4	68.5 ± 3.0	64.7 ± 1.6	0.290
BMI (kg/m^2^)	27.25 ± 0.34	28.37 ± 0.92	26.99 ± 0.35	0.113
eGFR (mL/min/1.73m^2^)	81.45 ± 3.99	67.09 ± 5.72	84.72 ± 4.60	0.086
Smoking history	24 (44.4)	0 (0.0)	24 (54.5)	0.001
Recent smoking (≤ 30 days)	5 (9.3)	0 (0.0)	5 (11.4)	0.571
Arterial hypertension	47 (87.0)	9 (90.0)	38 (86.4)	1.000
Diabetes	15 (27.8)	2 (20.0)	13 (29.5)	0.708
Dyslipidemia	47 (87.0)	8 (80.0)	39 (88.6)	0.601
PVD	11 (20.4)	2 (20.0)	9 (20.5)	1.000
Cerebrovascular disease	12 (22.2)	4 (40.0)	8 (18.2)	0.203
MI history	24 (44.4)	3 (30.0)	21 (47.7)	0.483
Recent MI (≤ 30 days)	14 (25.9)	2 (20.0)	12 (27.3)	1.000
Medication				
ACE inhibitor	21 (38.9)	1 (10.0)	20 (45.5)	0.069
ARB	14 (25.9)	1 (10.0)	13 (29.5)	0.263
β-blocker	35 (64.8)	5 (50.0)	30 (68.2)	0.297
CCB	10 (18.5)	1 (10.0)	9 (20.5)	0.667
Insulin	6 (11.1)	1 (10.0)	5 (11.4)	1.000
OHA	13 (24.1)	2 (20.0)	11 (25.0)	1.000
Nitrate	14 (25.9)	1 (10.0)	13 (29.5)	0.263

Abbreviations: ACE, angiotensin converting enzyme; ARB, angiotensin II receptor blocker; CCB, calcium channel blocker; CCS, Canadian Cardiovascular Society; eGFR, estimated glomerular filtration rate; MI, myocardial infarction.

### Histomorphologic characterization

**Fig 1:**
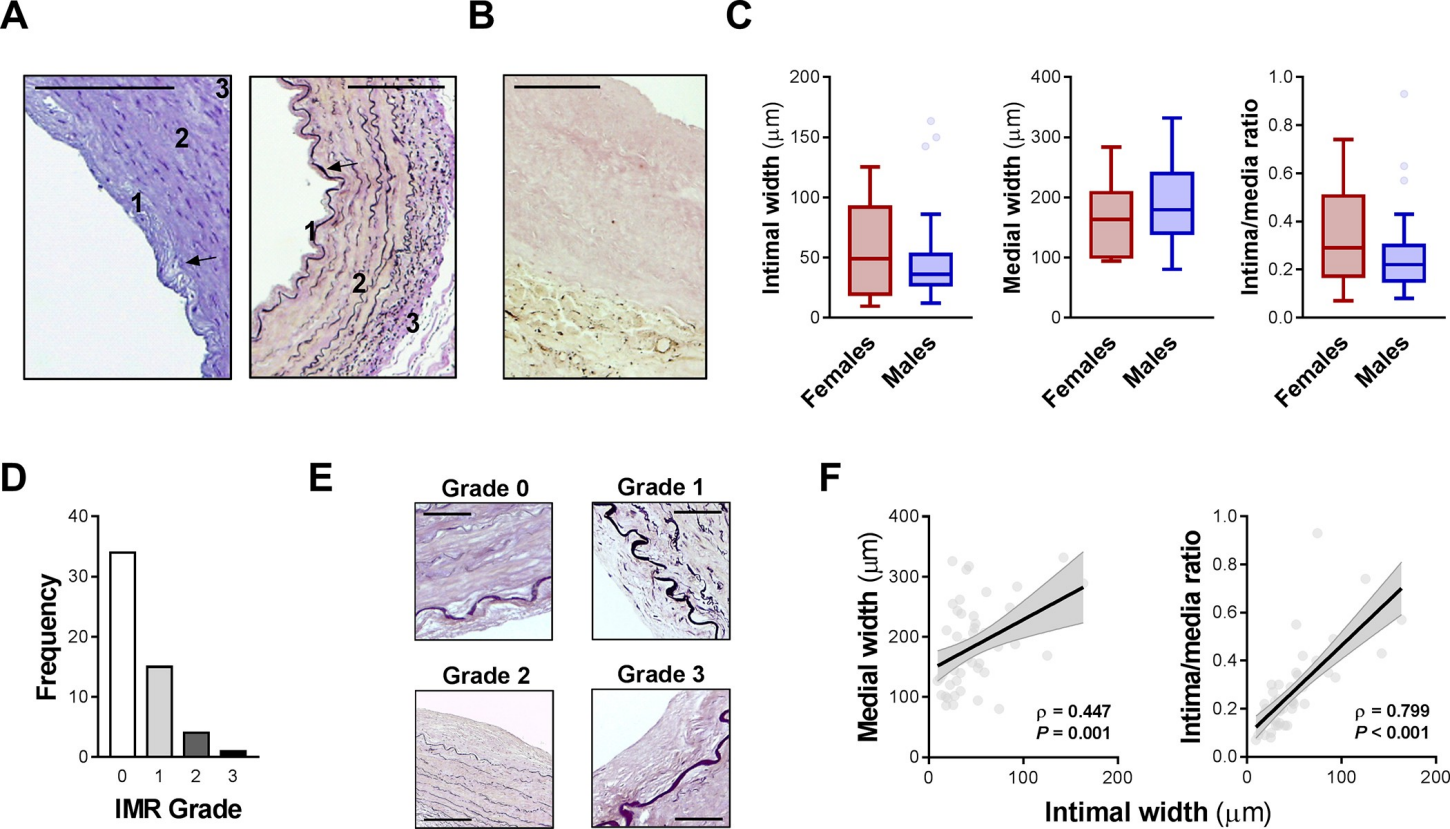
Histomorphology of human ITAs. (A) Histological observation shows the three vascular wall layers, tunica intima (1), tunica media (2) and tunica adventitia (3) by hematoxylin-eosin (left) and van Gieson (right) staining protocols (10x magnification). Scale bar represents 200 μm and arrows represent the internal elastic lamina. (B) Von Kossa (40x magnification) staining revealed predominant adventitial mild calcifications. Scale bar represents 200 μm. (C) Box plots for IW, MW and IMR according to gender. (D) Frequencies of IMR grades across the total population. (E) Representative photomicrographs for the IMR grades by van Gieson (10x magnification for grade 2 and 40x magnification for other grades) staining. Scale bar represents 200 μm in grade 2 and 50 μm for other grades. (F) Scatterplots of the correlation between histomorphometric parameters: IW vs MW and IMR.

All ITA samples presented characteristics of an elastic artery composed of three main layers: (a) tunica intima, (b) tunica media and (c) tunica adventitia, as presented in Fig 1A. Considering the classification proposed by Borović et al. [19], the tunica media presented from an elastomuscular to a muscular pattern, even though the musculoelastic and the muscular patterns were the most prevalent. No overt atherosclerotic lesion was seen. However, all samples presented mild calcifications observable through Von Kossa staining (Fig 1B). Considering the semi-quantitative classification proposed by Qureshi et al. [20] on the degree of calcification in inferior epigastric arteries determined by the Von Kossa staining method, mild adventitial calcifications were present in all samples. Furthermore, 53 (98.1%) presented minimal medial calcification and 50 (92.6%) minimal intimal calcification. In addition, we also observed several breaks in elastic laminas, especially in the internal elastic lamina in all samples.

As shown in Fig 1C, the mean IW was 47.95 ± 4.62 μm (range: 9.50 to 163.50 μm) and the mean MW was 184.54 ± 9.05 μm (range: 80.50 to 332.00 μm). The calculation of the IMR revealed a mean IMR of 0.27 ± 0.02 (range: 0.07 to 0.93). As can be seen in Table 2, no difference was observed according to gender.

**Table 2:** Histomorphologic parameters of total population and according to gender. Data presented as mean ± SEM.

Parameters	Total (n = 54)	Females (n = 10)	Males (n = 44)	P
IW (μm)	47.95 ± 4.62	54.33 ± 12.11	46.50 ± 5.00	0.516
MW (μm)	184.54 ± 9.05	163.70 ± 19.25	189.27 ± 10.17	0.276
IMR	0.27 ± 0.02	0.33 ± 0.07	0.25 ± 0.02	0.176

According to the classification proposed by Kaufer et al. [14], 34 (63.0%) patients did not present intimal hyperplasia, i.e. IMR ≤ 0.25 (Fig 1D). Also, 15 (27.8%) presented intimal hyperplasia of grade 1 (0.25 < IMR ≤ 0.5), 4 (7.4%) presented grade 2 (0.5 < IMR ≤ 0.75) and 1 (1.9%) presented grade 3 (IMR > 0.75). Representative photomicrographs for each IMR grade are presented in Fig 1E.

As presented in Fig 1F, results showed a significant correlation between IW and MW (Spearman’s ρ = 0.447; P = 0.001) and between IW and IMR (Spearman’s ρ = 0.799; P < 0.001), respectively. Moreover, MW did not significantly correlate with IMR (Spearman’s ρ = -0.101; P = 0.469).

### Histomorphology and clinical variables

Next, we aimed at identifying clinical variables associated with the histomorphologic parameters, i.e. IW, MW and IMR. Hence, we constructed multivariable linear regression models that showed several significant relationships as presented in Table 3. No multicollinearity issues were observed as the VIF was lower than 2 for all variables included in the models.

**Table 3:** Multivariable linear regression analysis of the association between clinical variables and histomorphometric parameters. Variance inflation factor (VIF) represents a measure of multicollinearity. Abbreviations: eGFR, estimated glomerular filtration rate.

Model	Variables	β	P	VIF
IW ^a^	Age (years)	0.35	0.031	1.64
	Gender	-0.22	0.098	1.11
	eGFR (mL/min/1.73m^2^)	0.49	0.005	1.87
	Myocardial infarction history	0.46	0.001	1.15
MW ^b^	eGFR (mL/min/1.73m^2^)	0.38	0.013	1.31
	Smoking history	-0.31	0.032	1.23
	Myocardial infarction history	0.24	0.085	1.13
	Nitrate therapy	0.28	0.047	1.13
IMR ^c^	Age (years)	0.51	0.001	1.73
	Gender	-0.29	0.019	1.11
	eGFR (mL/min/1.73m^2^)	0.35	0.035	1.94
	Recent smoking history (last 30 days)	0.32	0.012	1.13
	Arterial hypertension	-0.31	0.014	1.09
	Myocardial infarction history	0.27	0.034	1.18

^a^ Adjusted R^2^ = 0.194.

^b^ Adjusted R^2^ = 0.139.

^c^ Adjusted R^2^ = 0.304.

In regard to IW, a measure of intimal thickness, there was a positive association with (a) age (Spearman’s ρ = 0.159; P = 0.252) and (b) kidney function measured as eGFR (Spearman’s ρ = 0.026; P = 0.851) despite no significant correlations were seen. Although gender was also identified as predictor in the construction of the model, the association was not significant. Moreover, myocardial infarction history was associated with significantly increased IW (59.98±8.66 μm vs 38.33±3.93 μm, P = 0.030).

Concerning medial thickness, multivariable analysis showed a positive association with eGFR and nitrate medication and a negative association with smoking history. However, univariate analysis showed no significant difference or correlation between MW and these clinical variables.

Considering IMR, age (Spearman’s ρ = 0.292; P = 0.032) was positively and significantly associated with this histomorphometric parameter, whereas the correlation with eGFR was not significant (Spearman’s ρ = -0.113; P = 0.418). The associations with gender, recent smoking history, arterial hypertension and myocardial infarction where not confirmed by univariate analysis.

### Independent clinical predictors for intimal hyperplasia (IMR > 0.25)

**Fig 2:**
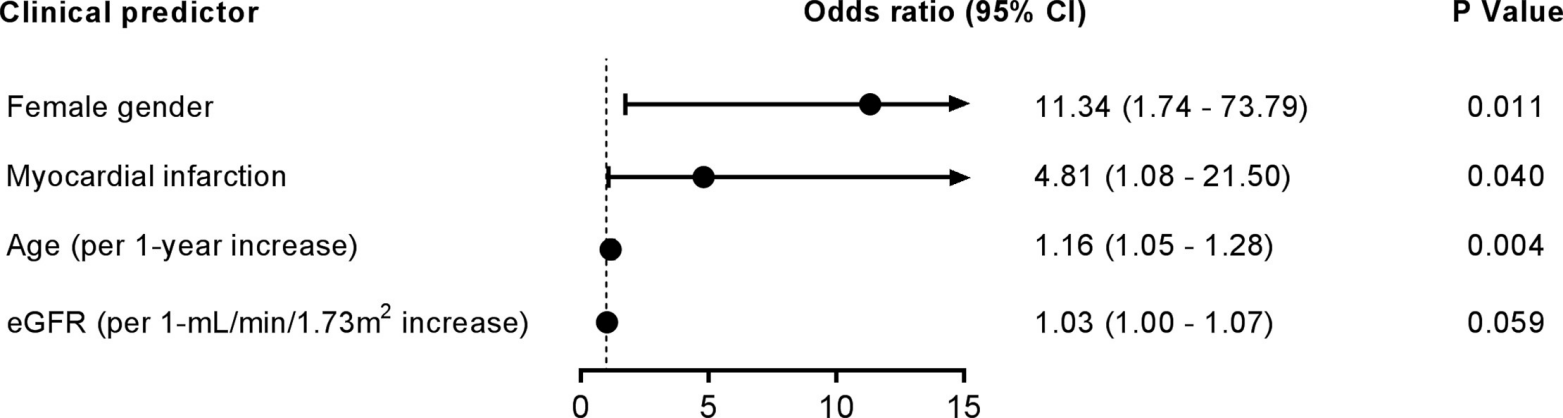
Forest plot for odds ratio for intimal hyperplasia (IMR > 0.25) in the total population. Results represent the odds ratio, 95% confidence intervals and P-value from multivariable logistic regression analysis. Dashed vertical line represents an odds ratio of 1. Abbreviations: CI, confidence interval; eGFR, estimated glomerular filtration rate.

Next, we aimed at identifying clinical predictors for intimal hyperplasia defined as IMR > 0.25 as proposed by Kaufer et al. [14]. Using multivariable logistic regression analysis, the following independent clinical predictors for intimal hyperplasia were identified: (a) age (odds ratio = 1.16, per 1-year increase), female gender (odds ratio = 11.34), kidney function measured as eGFR (odds ratio = 1.03, per 1-mL/min/1.73 m^2^ increase, non-significant) and myocardial infarction history (odds ratio = 4.81). As can be seen in Fig 2, all factors were significantly associated with increased predicted probability of intimal hyperplasia.

Relatively to the predictive performance of the model, 41.0% of the variability may be explained by the model (Nagelkerke R^2^ = 0.410) and it accurately predicts the probability of intimal hyperplasia (χ^2^ = 19.27, P = 0.001). The Hosmer-Lemeshow test for the model (χ^2^ = 11.59, P = 0.171) did not show significant differences between the observed and the predicted results and the ROC curve retrieved an AUC of 0.809 (95% confidence interval: 0.678 to 0.940), which is higher than the threshold of 0.700 proposed by Omar et al. [18]. Together, these results suggest that a good calibration and discriminatory power of the model.

## Discussion

Due to a higher functional and structural integrity, the human ITA has been recognized as a special vessel, which has been used in a wide array of studies, including as a model to study vascular physiology. Despite being considered an atherosclerosis-resistant vessel, several studies have emerged showing structural changes from intimal hyperplasia to overt atherosclerotic lesions, as previously reviewed by us [6]. Although distinct evidence has emerged regarding the relationship with risk factors, risk factors such as age [21, 22], arterial hypertension [21], diabetes mellitus [23], smoking [7] and chronic kidney disease [24] have been associated with structural changes such as intimal or medial thickening, increased IMR and/or others.

In regard to the histological observation, our study showed no overt atherosclerotic lesions. While this observation is in accordance with the majority of the previous reports, studies have also emerged showing atherosclerotic lesions in ITA samples [7, 21, 25].

Furthermore, intimal thickening assessed by histomorphometric analysis was observed in average. Also, mild calcifications were observed across the vascular wall layers in almost all samples. Although previous studies showed no medial calcifications in ITA samples [24, 26], Perrotta et al. [27] showed fragmented and discontinuous elastic lamellae oriented with areas of calcification in an ultrastructural study of the ITA. Together, these results suggest pre-atherosclerotic changes in ITA samples at the time of surgery.

Concerning the relationship between histomorphometric findings and the clinical profile, studies usually report univariate associations with traditional risk factors such as age, arterial hypertension or smoking. Taking into account the complexity and heterogeneity of risk factors and other clinical variables, we used multivariable linear regression analysis to identify clinical predictors of the histomorphometric parameters (IW, MW and IMR). As shown in Table 3, age, gender, kidney function expressed as eGFR and myocardial infarction were clinical predictors for IW and IMR, while eGFR was also a predictor for MW. Moreover, other clinical variables were associated with the several parameters.

Next, we used the IMR grade classification proposed by Kaufer et al. [14] and modified the groups into two main groups: (a) no intimal hyperplasia (IMR ≤ 0.25) and (b) intimal hyperplasia (IMR > 0.25). Accordingly, multivariable logistic regression analysis allowed the identification of several independent clinical predictors for intimal hyperplasia, namely: age, female gender, myocardial infarction. These results confirm the previous observation that these clinical variables were associated with IW and IMR.

Aging has long been considered a major non-modifiable cardiovascular risk factor that influences the vascular structure and function [28]. Previous studies have correlated aging with several vascular structure changes namely increased IW [7, 21, 24, 29, 30] and MW [30, 31] and IMR [21, 30]. Kinoshita et al. [24] also identified aging as an independent risk factor for intimal hyperplasia (odds ratio of 1.90 per 10-year increase).

In regard to female gender, no study has yet reported a significant association with intimal hyperplasia or other structural changes in ITAs [25, 30, 32]. However, important functional changes have been previously reported in ITAs from female patients compared to male patients [33, 34] which may be accompanied by structural changes.

Myocardial infarction has been previously associated with endothelial dysfunction in rat thoracic aortas [35]. To our knowledge, this is the first study to report such association, as previous studies have not typically reported the prevalence of this factor.

Kidney function expressed as eGFR also emerged as clinical predictor of intimal hyperplasia (odds ratio = 1.03). Although such association must be carefully interpreted due to the non-significant result (P = 0.059), this result is not in accordance with recent reports by Kinoshita et al. who showed that chronic kidney disease defined by a decreased eGFR at the time of surgery is associated both with intimal hyperplasia [24] and endothelial dysfunction [36].

Some limitations should be attributed to our study, namely the low sample size, the heterogeneity of risk factors and other clinical variables and the lack of ultrastructural assessment.

In conclusion, our study showed preatherosclerotic lesions in ITA samples from patients undergoing coronary revascularization and that intimal hyperplasia is associated not only with classical cardiovascular risk factors such as age and gender, but also with other clinical variables, namely kidney function and myocardial infarction history.
